# Supplementary material for: A novel systems biology approach to evaluate mouse models of late-onset Alzheimer’s disease
Source: Mol Neurodegener. 2020 Nov 10;15:67. doi: 10.1186/s13024-020-00412-5 (PMC7656729; doi:10.1186/s13024-020-00412-5)
Supplement: Supplementary file 1 — Additional file 1. [file 13024_2020_412_MOESM1_ESM.zip › Supplemental_Material_Preuss_et_al_09_28.docx]

**SUPPLEMENTAL MATERIAL**

**

**

**Supplemental Figure 1: Sequence divergence of genes prioritized for the nCounter Mouse AD panel compared to all transcripts within individual AMP-AD modules**

Within each of the 30 human co-expression modules, the selected key probes on the Mouse AD panel show a higher degree of sequence conservation when compared to the average transcript conservation level across AMP-AD modules.

**
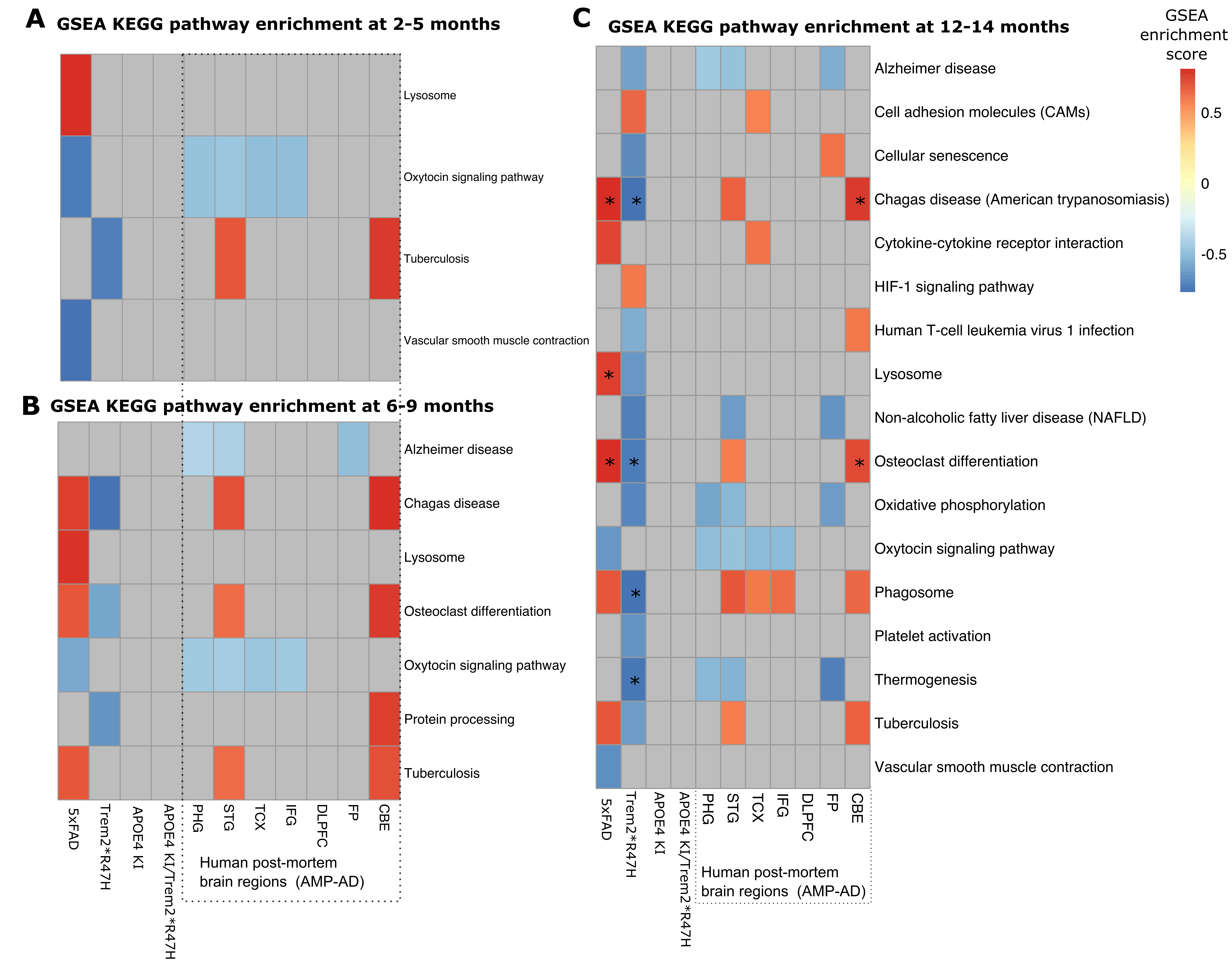
**

**Supplemental Figure 2: KEGG Gene Set Enrichment analysis (GSEA) comparing novel mouse models to human post-mortem brain regions**

Gene set enrichment was performed to identify significantly enriched molecular pathways from the KEGG database across multiple mouse models and age groups. Pathway enrichment was compared to seven human post-mortem brain regions from the AMP-AD cohorts. Significantly enriched pathways after multiple testing correction (adjusted Benjamini-Hochberg p < 0.05) are marked with an asterix (*). Up-regulated pathways are denoted in red. Down-regulated pathways are denoted in blue. Grey boxes symbolize pathways which did not show a significant enrichment across any of the novel mouse models or human post-mortem brain regions.


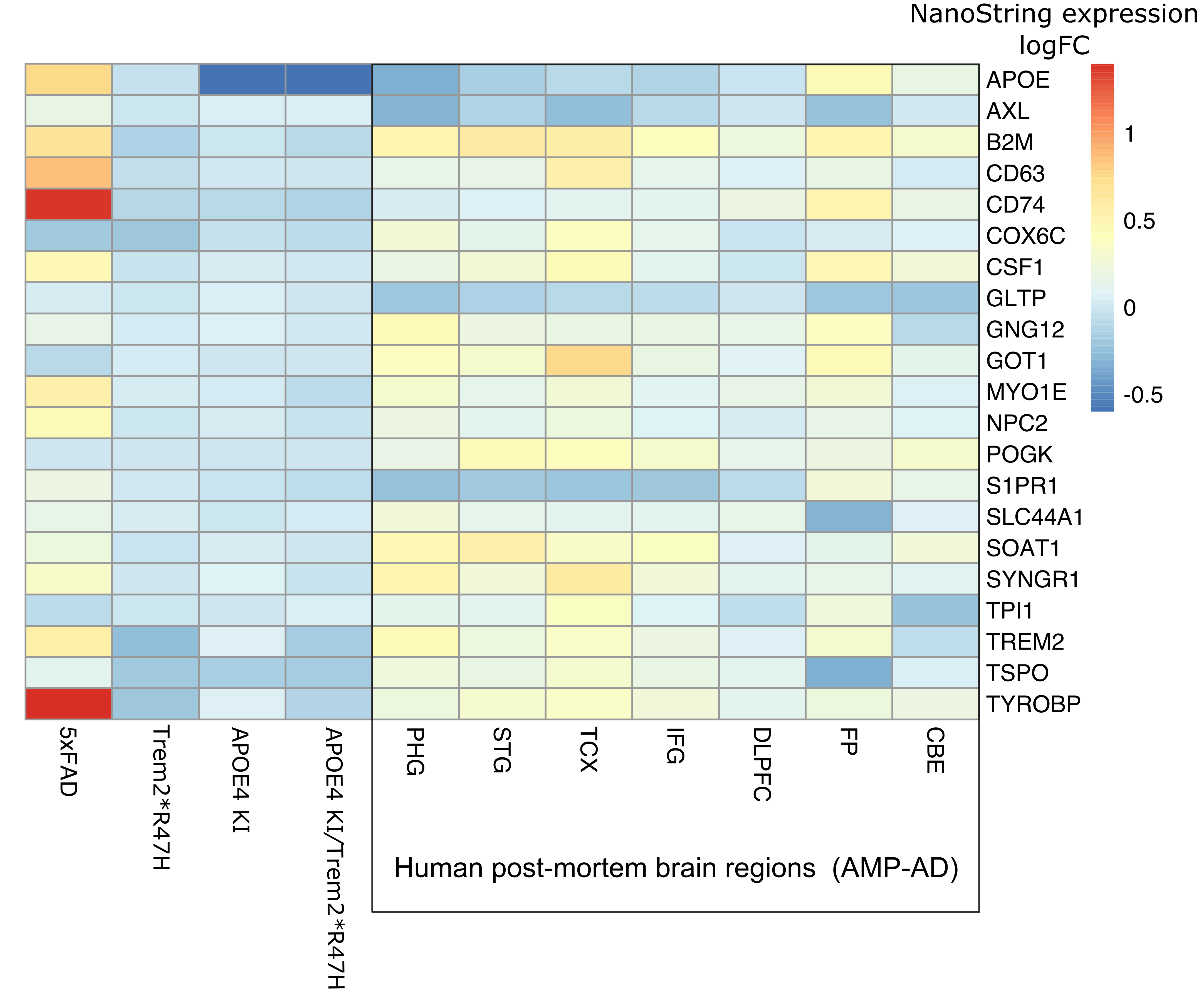


**Supplemental Figure 3: Disease associated microglia specific genes on the NanoString panel**

Expression signatures for disease associated microglia (DAM) from Keren-Shaul et al [1] are displayed in a heatmap. 5xFAD mice show a higher level of DAM activation when compared to B6 mice late in life (12-14 months). APOE4 KI, APOE4 KI/Trem2*R47H and Trem2*R47H mouse models show a lower level of expression when compared to age matched B6 mice and seven human post-mortem brain regions.

**
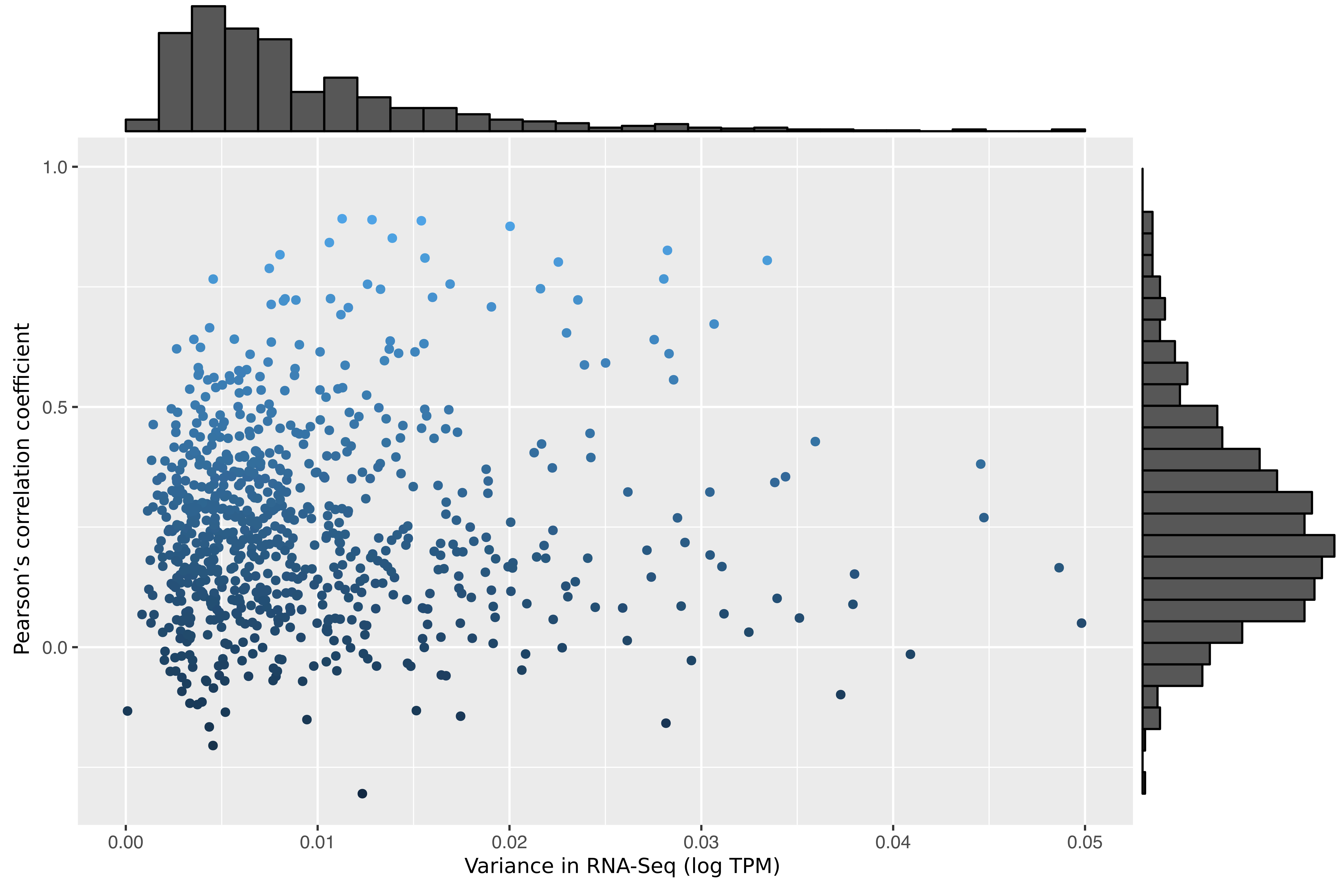
**

**Supplemental Figure 4: Comparison between Pearson’s correlation coefficients and variance in RNA-Seq expression for each probe across 137 samples**

The scatter plot depicts the correlation between the variance of each transcript measured by RNA-Seq versus the Pearson’s correlation coefficient between the NanoString and RNA-Seq expression for each probe across 137 samples. Of the 770 probes, 492 showed significant positive correlation (Pearson’s correlation coefficient > 0.17, p < 0.05), while 223 showed positive but insignificant correlation (0 < Pearson’s correlation coefficient < 0.17, p > 0). Two probes showed significant negative correlation (Pearson’s correlation coefficient < -0.20, p < 0) and 61 showed negative insignificant correlation (-0.30 < Pearson’s correlation coefficient < 0, p > 0). We did not observe any significant trend between variance and correlation coefficients.

**Supplemental Figure 5: Venn diagram for whole-genome sequencing results of the APOE4 KI/Trem2*R47H strain**

The number of genes harboring private deleterious variants in three individual founders of the APOE4 KI/Trem2*R47H strain for which whole-genome sequencing was performed. Three genes are shared across all founders, of which two genes are annotated as predicted genes (Gm11168, Gm10717) and Vmn2r115 which as an olfactory receptor.


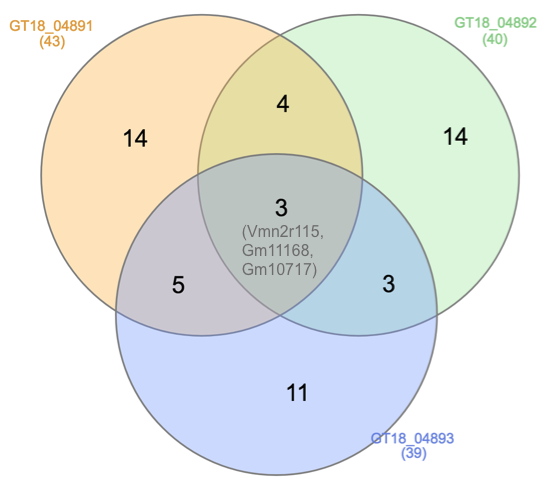


**SUPPLEMENT TABLE 1: Reactome pathway annotation for the five consensus clusters**

The table depicts the Reactome pathway annotations for the five functional consensus clusters associated with the 30 human co-expression modules. The five highest ranked and non-overlapping Reactome pathway annotations are highlighted and were used to annotate the five functional consensus clusters (A-E).

| **Consenus Cluster** | **Description** | **GeneRatio** | **p-value** | **qvalue** |
| --- | --- | --- | --- | --- |
| A | **Extracellular matrix organization** | 64/834 | 1.50E-06 | 1.39E-06 |
| A | Diseases associated with glycosaminoglycan metabolism | 16/834 | 1.50E-06 | 1.39E-06 |
| A | Diseases of glycosylation | 16/834 | 1.50E-06 | 1.39E-06 |
| A | Defective B4GALT7 causes EDS, progeroid type | 13/834 | 4.16E-06 | 3.88E-06 |
| A | Defective B3GAT3 causes JDSSDHD | 13/834 | 4.16E-06 | 3.88E-06 |
| B | **Immune System** | 311/1517 | 2.29E-13 | 1.97E-13 |
| B | Cytokine Signaling in Immune system | 123/1517 | 3.32E-13 | 2.87E-13 |
| B | Extracellular matrix organization | 113/1517 | 1.73E-13 | 1.49E-13 |
| B | Interferon gamma signaling | 51/1517 | 9.92E-14 | 8.55E-14 |
| B | Non-integrin membrane-ECM interactions | 38/1517 | 6.22E-12 | 5.36E-12 |
| C | **Neuronal System** | 178/2046 | 1.59E-30 | 1.30E-30 |
| C | Transmission across Chemical Synapses | 122/2046 | 1.22E-18 | 1.00E-18 |
| C | Neurotransmitter Receptor Binding And Downstream Transmission in the Postsynaptic Cell | 87/2046 | 2.28E-13 | 1.87E-13 |
| C | Potassium Channels | 65/2046 | 1.47E-10 | 1.20E-10 |
| C | Voltage gated Potassium channels | 37/2046 | 5.47E-11 | 4.47E-11 |
| D | Gene Expression | 271/1614 | 1.24E-05 | 1.06E-05 |
| D | **Cell Cycle, Mitotic** | 142/1614 | 5.34E-05 | 4.57E-05 |
| D | **Nonsense-Mediated Decay (NMD)** | 52/1614 | 2.49E-05 | 2.13E-05 |
| D | Nonsense Mediated Decay (NMD) enhanced by the Exon Junction Complex (EJC) | 52/1614 | 2.49E-05 | 2.13E-05 |
| D | Influenza Viral RNA Transcription and Replication | 52/1614 | 5.34E-05 | 4.57E-05 |
| E | Gene Expression | 517/2390 | 1.46E-55 | 1.01E-55 |
| E | **Organelle biogenesis and maintenance** | 178/2390 | 4.15E-19 | 2.89E-19 |
| E | **Cellular responses to stress** | 23/2390 | 1.45e-07 | 4.16e-05 |
| E | Cellular response to heat stress | 9/2390 | 4.23-06 | 5.57e-04 |

**SUPPLEMENT TABLE 2: Summary statistics nCoutner Mouse AD panel**

Summary statistics for the 30 human co-expression modules, including the annotation for brain regions and cohorts from which the specific modules were generated. In addition, NanoString probe coverage and functional consensus cluster membership are listed for each co-expression module.

| **Genes in AMP-AD module** | **Covered Nanostring**  **probes** | **Coverage [%]** | **Name** | **Brain Region** | **Cohort** | **Consensus Cluster** |
| --- | --- | --- | --- | --- | --- | --- |
| 1713 | 119 | 7 | TCXblue | temporal cortex | Mayo | A |
| 743 | 84 | 11 | IFGyellow | inferiorfrontal gyrus | Mount Sinai Brain Bank | A |
| 910 | 101 | 11 | PHGyellow | parahipocampal gyrus | Mount Sinai Brain Bank | A |
| 1751 | 183 | 10 | DLPFCblue | dorsolateral prefrontal cortex | ROS/MAP | B |
| 1977 | 200 | 10 | CBEturquoise | cerebellum | Mayo | B |
| 1131 | 78 | 7 | TCXturquoise | temporal cortex | Mayo | B |
| 1456 | 126 | 9 | IFGturquoise | inferiorfrontal gyrus | Mount Sinai Brain Bank | B |
| 1171 | 143 | 12 | STGblue | superiortemporal gyrus | Mount Sinai Brain Bank | B |
| 1195 | 104 | 9 | PHGturquoise | parahipocampal gyrus | Mount Sinai Brain Bank | B |
| 1001 | 107 | 11 | FPturquoise | frontal pole | Mount Sinai Brain bank | B |
| 3019 | 192 | 6 | DLPFCyellow | dorsolateral prefrontal cortex | ROS/MAP | C |
| 1739 | 157 | 9 | CBEyellow | cerebellum | Mayo | C |
| 2766 | 186 | 7 | TCXgreen | temporal cortex | Mayo | C |
| 4673 | 234 | 5 | IFGbrown | inferiorfrontal gyrus | Mount Sinai Brain Bank | C |
| 3414 | 211 | 6 | STGbrown | superiortemporal gyrus | Mount Sinai Brain Bank | C |
| 2123 | 165 | 8 | PHGbrown | parahipocampal gyrus | Mount Sinai Brain Bank | C |
| 4426 | 188 | 4 | FPyellow | frontal pole | Mount Sinai Brain Bank | C |
| 882 | 139 | 16 | DLPFCbrown | dorsolateral Prefrontal Cortex | ROS/MAP | D |
| 504 | 95 | 19 | CBEbrown | cerebellum | Mayo | D |
| 2013 | 151 | 8 | TCXyellow | temporal cortex | Mayo | D |
| 2885 | 236 | 8 | IFGblue | inferiorfrontal gyrus | Mount Sinai Brain Bank | D |
| 1799 | 159 | 9 | STGyellow | superiortemporal gyrus | Mount Sinai Brain Bank | D |
| 1151 | 139 | 12 | PHGgreen | parahipocampal gyrus | Mount Sinai Brain Bank | D |
| 1991 | 278 | 14 | FPblue | frontal pole | Mount Sinai Brain Bank | D |
| 2489 | 144 | 6 | DLPFCturquoise | dorsolateral prefrontal cortex | ROS/MAP | E |
| 4509 | 177 | 4 | CBEblue | cerebellum | Mayo | E |
| 1851 | 125 | 7 | TCXbrown | temporal cortex | Mayo | E |
| 2404 | 119 | 5 | STGturquoise | superiortemporal gyrus | Mount Sinai Brain Bank | E |
| 3733 | 170 | 5 | PHGblue | parahipocampal gyrus | Mount Sinai Brain Bank | E |
| 1289 | 76 | 6 | FPbrown | frontal pole | Mount Sinai Brain Bank | E |

**SUPPLEMENT TABLE 3: Annotations for selected NanoString probes covering human co-expression modules**

The attached table contains human gene to mouse probe annotations for the 770 selected probes on the nCounter Mouse AD panel (760 key genes and 10 housekeeping controls). Individual mouse probes were assigned to multiple human co-expression modules derived from different brain regions or cohorts. Gene scores indicate contribution of each probe set to relative module behavior for each assigned human co-expression module. AMP-AD drug targets as listed by Agora (agora.ampadportal.org) and the 10 housekeeping genes are highlighted (**See attached Excel table**).

**SUPPLEMENT TABLE 4: Overview of mouse samples used for strain survey**

| **Mouse model** | **Strain**  **Nomenclature (Number)** | **Total Samples** | **Sex** | **1-5 months** | **6-9 months** | **10-14 months** |
| --- | --- | --- | --- | --- | --- | --- |
|  |  |  |  |  |  |  |
| B6 | C57BL/6J  (JAX#664) | 35 | Female | 6 | 6 | 5 |
|  |  |  | Male | 5 | 6 | 7 |
| APOE4 KI | B6.Cg-Apoe^tm1.1(APOE*4)Adiuj^/J  (JAX # 27894) | 32 | Female | 6 | 6 | 5 |
|  |  |  | Male | 5 | 6 | 4 |
| Trem2*R47H | B6.Cg-Apoe^tm1.1(APOE*4)Adiuj^Trem2^em1Adiuj^/J (JAX #27918) | 35 | Female | 5 | 6 | 6 |
|  |  |  | Male | 6 | 6 | 6 |
| APOE4 KI/Trem2*R47H | B6.Cg-Apoe^tm1.1(APOE*4)Adiuj^Trem2^em1Adiuj^/J (JAX #28709) | 35 | Female | 6 | 6 | 5 |
|  |  |  | Male | 5 | 6 | 7 |
| 5xFAD | B6SJL-Tg(APPSwFlLon,PSEN1*M146L*L286V)6799Vas/Mmjax | 38 | Female | 13 | 3 | 3 |
|  |  |  | Male | 13 | 3 | 3 |

**SUPPLEMENT TABLE 5: Inventory of differential expression analysis between three novel LOAD mouse models using the NanoString Mouse AD panel**

The number of differentially expressed genes after FDR adjusted p-value cutoff of 0.05 between each mouse model and aged matched mice are highlighted. Genes with the strongest log fold changes are listed for each time point and mouse model respectively.

|  | **APOE4 KI vs. C57BL/6J** | | **Trem2*R47H vs. C57BL/6J** | | **APOE4 KI/Trem2*R47H**  **vs. C57BL/6J** | | **APOE4 KI vs.**  **Trem2*R47H** | |
| --- | --- | --- | --- | --- | --- | --- | --- | --- |
|  | **Up** | **Down** | **Up** | **Down** | **Up** | **Down** | **Up** | **Down** |
| 2-5 months  (FDR adjusted p-value cutoff of 0.05) | 0 | 1 | 0 | 0 | 0 | 1 | 0 | 1 |
| Genes with  logFC < -1 or > 1 | None | Apoe  (mouse) | None | None | None | Apoe  (mouse) | None | Apoe  (mouse) |
| 6-9 months  (FDR adjusted p-value cutoff of 0.05) | 4 | 6 | 18 | 15 | 50 | 30 | 18 | 11 |
| Genes with  logFC < -1 or > 1 | None | Apoe  (mouse) | None | None | None | Apoe  (mouse) | None | None |
| 10-14 months  (FDR adjusted p-value cutoff of 0.05) | 0 | 1 | 0 | 7 | 0 | 5 | 2 | 8 |
| Genes with  logFC < -1 or > 1 | None | Apoe  (mouse) | None | None | None | Apoe  (mouse) | None | None |
|  | A detailed overview of differential expression results for the 760 NanoString key probes and each time point across the three different strains compared to B6 controls can be found in supplemental table 6 | | | | | | A detailed overview of differential expression results for 760 NanoString key probes between APOE4 KI and Trem2*R47H mice can be found in supplemental table 7 | |

**SUPPLEMENT TABLE 6: Results from differential gene expression analysis for the APOE4 KI, Trem2*R47H and APOE4 KI/Trem2*R47H models compared to aged matched C57BL/6J mice**

The table contains the results for the differential gene expression analysis for the three novel LOAD models for three timepoints (2-5, 6-9, 10-14 months). Samples are compared to age matched B6 mice. Differentially expressed genes were determined using FDR adjusted p-value cutoffs of p < 0.05. (**See attached Excel table**).

**SUPPLEMENT TABLE 7: Results from differential gene expression analysis between APOE4 KI and aged matched Trem2*R47H mice**

The table contains the results for the differential gene expression analysis between the APOE4 KI mouse model and the Trem2*R47H model for three timepoints (2-5, 6-9, 10-14 months). Differentially expressed genes were determined using FDR adjusted p-value cutoffs of p < 0.05. (**See attached Excel table**).

**SUPPLEMENT TABLE 8: Functional annotations for private deleterious variants identified by whole-genome sequencing in three founders of the APOE4 KI/Trem2*R47H strain**

Functional annotations of high confidence deleterious variants identified by whole-genome sequencing in three independent founders of the APOE4 KI/Trem2*R47H strain. Annotations are provided for the mm10 genome build. Variants were filtered based on genotype quality (Genotype Quality > 50) and deleterious effects on the protein coding level (missense, stop-gain, frame-shift, splicing).

| CHR.  (mm10) | POSITION  (mm10) | REF | ALT | SNP  FUNCTIONAL  CLASS | GENE  NAME | AMINO  ACID  CHANGE | FOUNDER |
| --- | --- | --- | --- | --- | --- | --- | --- |
| chr10 | 80534525 | G | T | MISSENSE | Atp8b3 | S90Y | GT_18_04891 |
| chr11 | 77134911 | C | T | MISSENSE | Efcab5 | G662E | GT_18_04891 |
| chr13 | 12997464 | A | C | MISSENSE | Prl2c2 | S107A | GT_18_04891 |
| chr14 | 51411022 | A | T | MISSENSE | Vmn2r88 | T8S | GT_18_04891 |
| chr16 | 32754911 | G | T | MISSENSE | Muc4 | S1595I | GT_18_04891 |
| chr16 | 32754925 | G | C | MISSENSE | Muc4 | A1600P | GT_18_04891 |
| chr17 | 23346264 | C | T | MISSENSE | Vmn2r115 | S375F | GT_18_04891 |
| chr17 | 23477858 | G | A | MISSENSE | Vmn2r117 | H192Y | GT_18_04891 |
| chr17 | 23477897 | G | A | MISSENSE | Vmn2r117 | H179Y | GT_18_04891 |
| chr17 | 23477909 | C | A | MISSENSE | Vmn2r117 | V175F | GT_18_04891 |
| chr17 | 23477921 | G | T | MISSENSE | Vmn2r117 | P171T | GT_18_04891 |
| chr17 | 23477939 | G | A | MISSENSE | Vmn2r117 | L165F | GT_18_04891 |
| chr17 | 25654471 | G | A | MISSENSE | Lmf1 | V317M | GT_18_04891 |
| chr3 | 15416972 | T | G | MISSENSE | Sirpb1a | M99L | GT_18_04891 |
| chr3 | 15416977 | T | C | MISSENSE | Sirpb1a | N97S | GT_18_04891 |
| chr3 | 15416986 | G | T | MISSENSE | Sirpb1a | T94K | GT_18_04891 |
| chr4 | 146196340 | T | C | MISSENSE | Zfp600 | I526T | GT_18_04891 |
| chr4 | 147058728 | C | T | MISSENSE | Rex2 | P558S | GT_18_04891 |
| chr4 | 147678316 | A | C | MISSENSE | Zfp534 | L20V | GT_18_04891 |
| chr4 | 147755125 | C | A | MISSENSE | Gm13157 | S423I | GT_18_04891 |
| chr5 | 14914797 | A | C | MISSENSE | Gm9758 | F11L | GT_18_04891 |
| chr5 | 26021746 | T | G | MISSENSE | Gm5862 | T53P | GT_18_04891 |
| chr5 | 26105214 | C | G | MISSENSE | 5031410I06Rik | K26N | GT_18_04891 |
| chr6 | 128359192 | G | A | MISSENSE | Rhno1 | H27Y | GT_18_04891 |
| chr8 | 19693826 | C | T | NONSENSE | Gm6483 | Q156* | GT_18_04891 |
| chr8 | 20634946 | T | A | MISSENSE | Gm21119 | F285I | GT_18_04891 |
| chr8 | 94521411 | G | A | MISSENSE | Nlrc5 | R1669H | GT_18_04891 |
| chr9 | 3005128 | T | G | MISSENSE | Gm11168 | S202R | GT_18_04891 |
| chr9 | 3005204 | T | C | MISSENSE | Gm11168 | C16R | GT_18_04891 |
| chr9 | 3013264 | C | A | MISSENSE | Gm10721 | L41I | GT_18_04891 |
| chr9 | 3014034 | G | A | MISSENSE | Gm10721 | G142S | GT_18_04891 |
| chr9 | 3017510 | T | A | MISSENSE | Gm10719 | F33L | GT_18_04891 |
| chr9 | 3017522 | G | T | MISSENSE | Gm10719 | K37N | GT_18_04891 |
| chr9 | 3017602 | G | C | MISSENSE | Gm10719 | R64T | GT_18_04891 |
| chr9 | 3018962 | T | C | MISSENSE | Gm10719 | L69S | GT_18_04891 |
| chr9 | 3020588 | C | T | MISSENSE | Gm10719 | S145L | GT_18_04891 |
| chr9 | 3020835 | T | A | MISSENSE | Gm10719 | F207I | GT_18_04891 |
| chr9 | 3023936 | A | T | MISSENSE | Gm10718 | H129L | GT_18_04891 |
| chr9 | 3027920 | A | G | MISSENSE | Gm10717 | S132G | GT_18_04891 |
| chr9 | 3028058 | T | A | MISSENSE | Gm10717 | Y178N | GT_18_04891 |
| chr9 | 3030919 | T | A | NONSENSE | Gm10717 | C116* | GT_18_04891 |
| chr9 | 3030939 | T | A | MISSENSE | Gm10717 | V123D | GT_18_04891 |
| chr9 | 3035002 | A | T | MISSENSE | Gm17535 | T134S | GT_18_04891 |
| chr11 | 99792794 | A | G | MISSENSE | Gm11596 | S167P | GT_18_04892 |
| chr13 | 107889578 | G | GC | NONSENSE | Zswim6 | A157A? | GT_18_04892 |
| chr14 | 51452167 | G | C | MISSENSE | Vmn2r89 | L42F | GT_18_04892 |
| chr14 | 51456033 | T | C | MISSENSE | Vmn2r89 | V280A | GT_18_04892 |
| chr14 | 51456477 | T | C | MISSENSE | Vmn2r89 | V428A | GT_18_04892 |
| chr16 | 32753450 | T | C | MISSENSE | Muc4 | S1109P | GT_18_04892 |
| chr16 | 32753451 | C | T | MISSENSE | Muc4 | S1109F | GT_18_04892 |
| chr16 | 32753508 | C | A | MISSENSE | Muc4 | T1128K | GT_18_04892 |
| chr16 | 32753640 | C | A | MISSENSE | Muc4 | P1172Q | GT_18_04892 |
| chr16 | 32753645 | A | T | MISSENSE | Muc4 | T1174S | GT_18_04892 |
| chr17 | 23359233 | T | C | MISSENSE | Vmn2r115 | L560P | GT_18_04892 |
| chr17 | 35620809 | C | G | MISSENSE | Gm9573 | R828S | GT_18_04892 |
| chr1 | 85642622 | G | T | MISSENSE | Sp140 | R420M | GT_18_04892 |
| chr1 | 88306678 | G | A | NONSENSE | Trpm8 |  | GT_18_04892 |
| chr1 | 164193733 | T | C | MISSENSE | F5 | L1259P | GT_18_04892 |
| chr2 | 6046989 | G | A | MISSENSE | Upf2 | G1154D | GT_18_04892 |
| chr4 | 147057847 | G | T | MISSENSE | Rex2 | C264F | GT_18_04892 |
| chr4 | 147492443 | A | C | NONSENSE | Gm13152 |  | GT_18_04892 |
| chr4 | 156334454 | G | A | MISSENSE | Vmn2r-ps159 | V344I | GT_18_04892 |
| chr4 | 156334455 | T | A | MISSENSE | Vmn2r-ps159 | V344E | GT_18_04892 |
| chr5 | 14911434 | G | C | MISSENSE | Gm9758 | A177G | GT_18_04892 |
| chr5 | 14935772 | T | G | MISSENSE | Speer4e | K134Q | GT_18_04892 |
| chr5 | 25949868 | G | T | MISSENSE | Gm21671 | H245N | GT_18_04892 |
| chr5 | 27497106 | C | A | MISSENSE | Speer4b | V219L | GT_18_04892 |
| chr6 | 128357907 | G | A | MISSENSE | Rhno1 | A151V | GT_18_04892 |
| chr6 | 128357908 | C | T | MISSENSE | Rhno1 | A151T | GT_18_04892 |
| chr6 | 132207782 | A | T | MISSENSE | Prb1 | M296K | GT_18_04892 |
| chr6 | 132207783 | T | G | MISSENSE | Prb1 | M296L | GT_18_04892 |
| chr7 | 22163170 | A | G | MISSENSE | Vmn1r142 | L289P | GT_18_04892 |
| chr9 | 3004984 | A | C | MISSENSE | Gm11168 | K175T | GT_18_04892 |
| chr9 | 3006288 | A | C | MISSENSE | Gm11168 | T103P | GT_18_04892 |
| chr9 | 3016626 | C | T | MISSENSE | Gm10720 | A109V | GT_18_04892 |
| chr9 | 3016649 | G | C | MISSENSE | Gm10720 | V117L | GT_18_04892 |
| chr9 | 3016674 | A | G | MISSENSE | Gm10720 | Q125R | GT_18_04892 |
| chr9 | 3016675 | G | T | MISSENSE | Gm10720 | Q125H | GT_18_04892 |
| chr9 | 3025874 | T | G | MISSENSE | Gm10717 | V153G | GT_18_04892 |
| chr9 | 3028694 | A | T | MISSENSE | Gm10717 | K216M | GT_18_04892 |
| chr9 | 3032579 | C | G | MISSENSE | Gm10717 | T164S | GT_18_04892 |
| chr9 | 3032588 | C | T | MISSENSE | Gm10717 | A167V | GT_18_04892 |
| chr9 | 3038268 | A | T | MISSENSE | Gm10715 | Q211H | GT_18_04892 |
| chr14 | 19415942 | A | G | MISSENSE | Gm21738 | L199S | GT18_04893 |
| chr14 | 51414513 | T | C | MISSENSE | Vmn2r88 | V436A | GT18_04893 |
| chr17 | 22618331 | C | T | MISSENSE | Vmn2r112 | S591F | GT18_04893 |
| chr17 | 22618336 | C | A | MISSENSE | Vmn2r112 | L593I | GT18_04893 |
| chr17 | 22618354 | G | T | MISSENSE | Vmn2r112 | A599S | GT18_04893 |
| chr17 | 23359533 | T | C | MISSENSE | Vmn2r115 | I660T | GT18_04893 |
| chr17 | 23460386 | G | C | MISSENSE | Vmn2r117 | N621K | GT18_04893 |
| chr17 | 23477294 | C | T | MISSENSE | Vmn2r117 | E380K | GT18_04893 |
| chr17 | 23477308 | G | A | MISSENSE | Vmn2r117 | S375F | GT18_04893 |
| chr3 | 93397493 | T | A | MISSENSE | Rptn | L711Q | GT18_04893 |
| chr4 | 145537455 | A | T | MISSENSE | Gm13225 | N341I | GT18_04893 |
| chr4 | 145537473 | C | A | MISSENSE | Gm13225 | T347K | GT18_04893 |
| chr4 | 146124988 | G | A | MISSENSE | Gm13051 | G330D | GT18_04893 |
| chr4 | 147332245 | A | C | MISSENSE | Gm13151 | T379P | GT18_04893 |
| chr4 | 156334562 | T | G | MISSENSE | Vmn2r-ps159 | S380A | GT18_04893 |
| chr5 | 26019348 | G | T | MISSENSE | Gm5862 | H208N | GT18_04893 |
| chr5 | 26057346 | T | G | MISSENSE | Gm7347 | K59T | GT18_04893 |
| chr5 | 26057353 | G | T | MISSENSE | Gm7347 | P57T | GT18_04893 |
| chr5 | 26057365 | T | A | MISSENSE | Gm7347 | T53S | GT18_04893 |
| chr5 | 129020892 | C | T | MISSENSE | Ran | L50F | GT18_04893 |
| chr5 | 129020895 | G | A | MISSENSE | Ran | V51I | GT18_04893 |
| chr5 | 129020973 | G | A | MISSENSE | Ran | D77N | GT18_04893 |
| chr7 | 38867419 | T | G | MISSENSE | Gm6605 | E195A | GT18_04893 |
| chr9 | 3006540 | A | G | NONSENSE | Gm11168 |  | GT18_04893 |
| chr9 | 3013508 | G | A | MISSENSE | Gm10721 | R122H | GT18_04893 |
| chr9 | 3017110 | A | T | MISSENSE | Gm10720 | Y190F | GT18_04893 |
| chr9 | 3018898 | G | T | MISSENSE | Gm10719 | V48F | GT18_04893 |
| chr9 | 3021580 | T | A | MISSENSE | Gm10719 | F223Y | GT18_04893 |
| chr9 | 3021589 | C | A | MISSENSE | Gm10719 | S226Y | GT18_04893 |
| chr9 | 3025485 | A | AT | NONSENSE | Gm10717 | F24F? | GT18_04893 |
| chr9 | 3027600 | C | A | MISSENSE | Gm10717 | P25H | GT18_04893 |
| chr9 | 3032275 | T | C | MISSENSE | Gm10717 | S63P | GT18_04893 |
| chr9 | 3036916 | A | T | MISSENSE | Gm10715 | I14F | GT18_04893 |
| chr9 | 3037267 | G | T | MISSENSE | Gm10715 | Q129H | GT18_04893 |
| chr9 | 3037304 | C | A | MISSENSE | Gm10715 | R142S | GT18_04893 |
| chr9 | 3037815 | G | T | MISSENSE | Gm10715 | M214I | GT18_04893 |
| chrX | 170674746 | C | A | MISSENSE | Asmt | D128E | GT18_04893 |
| chrX | 170674747 | G | T | MISSENSE | Asmt | G129C | GT18_04893 |
| chrY | 5249036 | A | G | MISSENSE | Gm21820 | C136R | GT18_04893 |

References:

1. Keren-Shaul H, Spinrad A, Weiner A, Matcovitch-Natan O, Dvir-Szternfeld R, Ulland TK, et al. A Unique Microglia Type Associated with Restricting Development of Alzheimer’s Disease. Cell. 2017;169: 1276–1290.e17. doi:10.1016/j.cell.2017.05.018
